# Navitoclax (ABT263) reduces inflammation and promotes chondrogenic phenotype by clearing senescent osteoarthritic chondrocytes in osteoarthritis

**DOI:** 10.18632/aging.103177

**Published:** 2020-07-01

**Authors:** Hao Yang, Cheng Chen, Hao Chen, Xiaojun Duan, Juan Li, Yi Zhou, Weinan Zeng, Liu Yang

**Affiliations:** 1 Center for Joint Surgery, Southwest Hospital, Third Military Medical University, Army Medical University, Chongqing 400038, People’s Republic of China; 2 Department of Orthopedics, West China Hospital, West China School of Medicine, Sichuan University, Chengdu 610041, People’s Republic of China

**Keywords:** osteoarthritis, inflammation, cellular senescence, senolytic, chondrocytes

## Abstract

Cell senescence is a chronic process associated with age-related degenerative diseases such as osteoarthritis (OA). Senescent cells (SnCs) accumulate in the articular cartilage and synovium, leading to OA pathologies. The accumulation of SnCs in the cartilage results in a senescence-associated secretory phenotype (SASP) and age-related inflammation and dysfunction. Selective removal of SnCs by senolytic agent as a therapeutic strategy has been developed recently. In this study, we examined the ability of the senolytic drug ABT263 (navitoclax) to clear SnCs and further evaluated the therapeutic effect of ABT263 on post-traumatic OA. Monolayer and 3D pellet cultured osteoarthritic chondrocytes were used to evaluate the effect of ABT263 in vitro and a DMM rat model was established for in vivo experiments. We found that ABT263 reduced the expression of inflammatory cytokines and promoted cartilage matrix aggregation in OA chondrocyte pellet culture by inducing SnC apoptosis. Moreover, OA pathological changes in the cartilage and subchondral bone in post-traumatic OA rat were alleviated by ABT263 intra-articular injection. These results demonstrated that ABT263 not only improves inflammatory microenvironment but also promotes cartilage phenotype maintenance in vitro. Furthermore, ABT263 might play a protective role against post-traumatic OA development. Therefore, strategies targeting SnC elimination might be promising for the clinical therapy of OA.

## INTRODUCTION

Osteoarthritis (OA) is the most common chronic joint disease and is becoming more prevalent worldwide with increasing life expectancy and aging of populations [1]. It is thought to be a multifactorial degenerative joint disease, resulting in excessive morbidity, physical disability, reduced life quality, and extra health costs [2, 3]. Several risk factors have been associated with OA onset and development, including age, gender, genetic predisposition, obesity, trauma, and several biochemical and biomechanical components [4, 5]. Among them, aging is considered an important pathological factor and a leading predictor of OA. However, the mechanisms linking between aging and OA pathogenesis remain unclear [5–7]. Previous studies demonstrated that moderate physical activity and supplementation of vitamin D as a non-pharmacologic treatment for aging cartilage could ameliorate cartilage degeneration in early stage of OA and have a favorable impact on cartilage thickness development [8–10]. Several cellular and molecular hallmarks of aging have been reported to be imminent causes of age-related dysfunction in the knee joint, including age-related inflammation and cellular senescence [5].

Cell senescence is characterized by arrest of cell cycle, changes in metabolism, and loss of proliferative ability [11]. Various markers have been used to identify senescent cells (SnCs), including gene expression of *p21*, *p16*, and *p53*, elevated levels of reactive oxygen species (ROS), and activation of senescence-associated β-galactosidase (SA-β-Gal) [12, 13]. In aging articular cartilage, the senescent-related alterations in chondrocytes and mesenchymal stem cells (MSCs) during OA, such as hypertrophy and loss of cell proliferative and differentiation capacity, may affect chondrogenic differentiation of MSCs and bring obstruction to cartilage regeneration [14]. In this regard, the term “chondrosenescence” was proposed to describe the age-dependent destruction of chondrocytes and highlight its hallmarks, and explain how they affect the phenotype of these cells and their specialized functions [15]. Furthermore, SnCs have been shown accumulation in OA cartilage tissues with aging [16]. These SnCs exhibit positive staining of SA-β-Gal, increased level of the senescence-related gene *p16INK4a* (also known as cyclin-dependent kinase inhibitor 2a; Cdkn2a) [17–19], senescence-associated secretory phenotype (SASP), increased production of pro-inflammatory mediators, and increased secretion of cytokines and chemokines [20, 21]. Selective removal of these SnCs through a senolytic molecule (UBX0101) from OA chondrocytes has been shown to reduce the expression of inflammation- and age-related molecules, and simultaneously delay the progression of post-traumatic OA in p16–3MR mice [22]. This finding supports a promising therapeutic strategy by targeting SnCs for OA treatment.

Another senolytic pharmacological agent navitoclax (also named ABT263), a specific inhibitor of the BCL-2 and BCL-xL proteins, has been reported to selectively clear SnCs in the hematopoietic system from premature aging mice after total-body irradiation by inducing cell apoptosis, and thus, rejuvenating aged tissue stem cells in normally aged mice [23, 24]. In this study, we investigated whether ABT263 could selectively eliminate SnCs in OA tissue. We first verified the clearance effect of SnCs by ABT263 in ionizing radiation (IR)-induced rat knee chondrocytes in vitro. We then measured the expression of SASP-associated proteins and chondrogenic differentiation markers after treatment with ABT263. Furthermore, we investigated whether intra-articular injection of ABT263 could attenuate the progression of post-traumatic OA and alleviate the cartilage damage in Sprague–Dawley (SD) rat knees.

## RESULTS

### ABT263 exhibited a dose-dependent manner clearance effect in IR-induced SnCs

**Figure 1:**
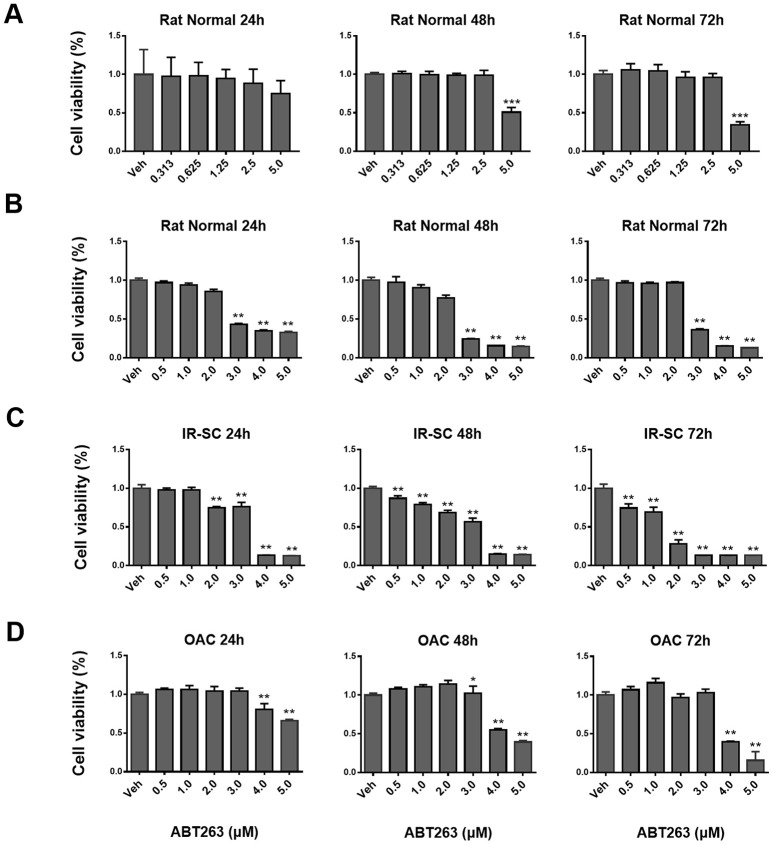
**The effect of ABT263 on the viability of ionizing radiation-induced senescent rat chondrocytes (IR-SC), normal rat chondrocytes (NC) and human OA chondrocytes in culture.** (**A**) Quantification of viable NC at 24, 48, and 72 h after treatment with increasing concentrations of ABT263. (**B**) CCK8 assay further confirmed that ABT263 showed cytotoxicity to NC after treatment with ABT263 (> 3 μM). (**C**) CCK8 tests showed a dose-dependent effect of ABT263 on killing IR–SC. (**D**) Quantification of viable P1 human OA chondrocytes after treatment with increasing concentrations of ABT263 for 24, 48, and 72 h. Data were shown as mean ± standard deviation. N = 3 per group.

To test whether the isolated cells were chondrocytes, safranin O, toluidine blue, and immunohistochemical (IHC) staining of collagen type II and aggrecan were performed. Positive staining results verified that chondrocytes were successfully isolated and cultured in vitro (Supplementary Figure 1). The CCK-8 assay was performed after treatment with ABT263. The results showed that ABT263 did not exert cytotoxicity on normal rat chondrocytes at concentrations less than 2.5 μM, whereas the cytotoxic effect appeared when the ABT263 concentration was higher than 3 μM (Figure 1A, 1B). The results presented in Figure 1C showed that ABT263 had a dose-dependent clearance effect on IR-induced senescent rat chondrocytes, indicating that the clearance efficiency of ABT263 on SnCs is obvious. For human OA chondrocytes, the results presented no statistical significance with 3 μM ABT263 treatment for 24 and 72 h, suggesting that ABT263 has no apparent cytotoxicity at an appropriate concentration (Figure 1D).

**Figure 2:**
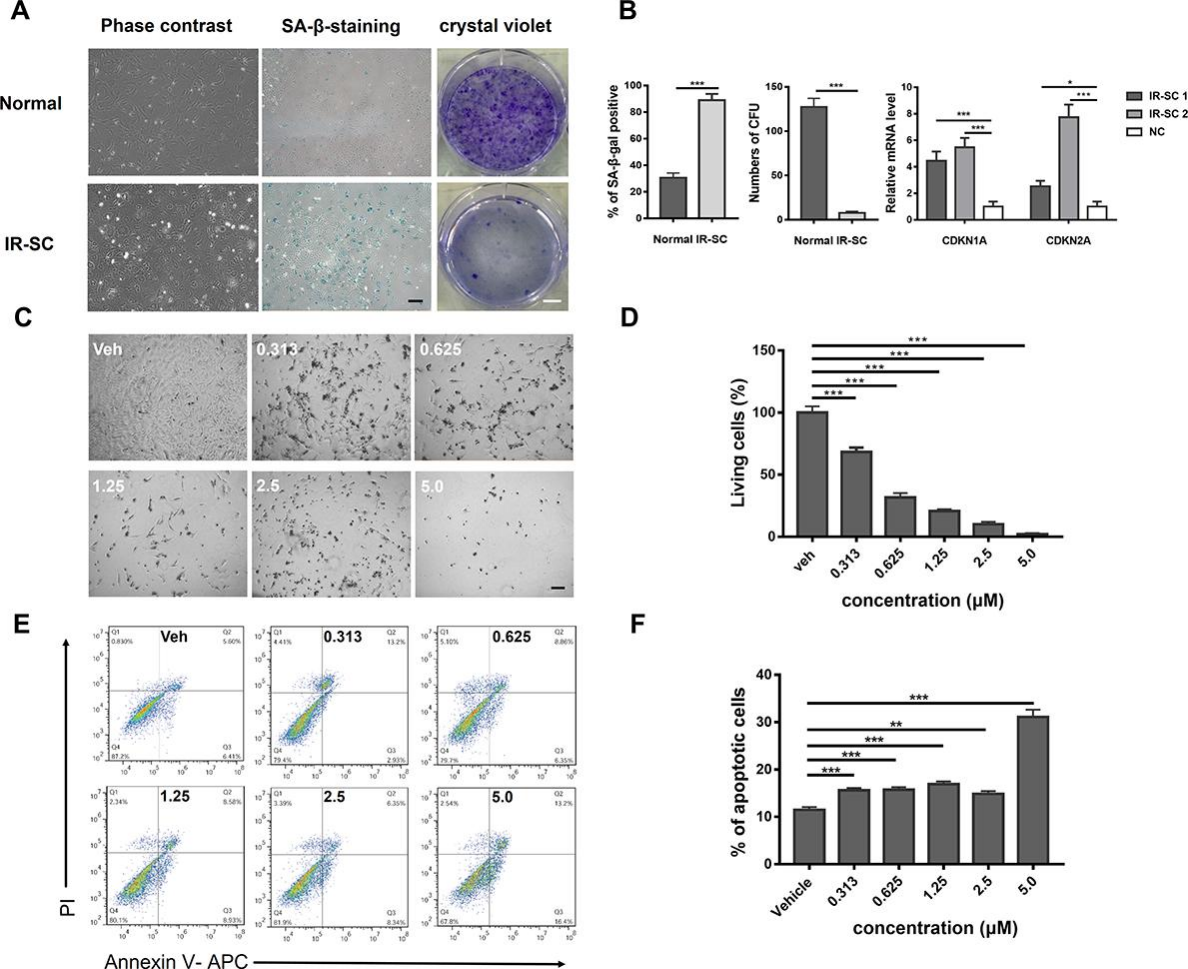
**Induction of senescent rat chondrocytes by ionization irradiation and dose-dependent killing effect of ABT263 on IR-SC via apoptosis.** (**A**) Morphology and crystal violet staining of senescent rat chondrocytes and normal rat chondrocytes. (**B**) Quantitative analysis of SA–β–gal staining and crystal violet staining. IR-SC 1 and IR-SC 2 were independently cultured IR-SCs generated from different batches of ionization irradiation under same circumstance. Data were presented as mean ± standard deviation. N = 3 per group. of fold changes from 3 independent experiments. **P < 0.01 and ***P < 0.001 vs. NC by unpaired t-test. (**C**) Representative images of IR-SC in culture after incubation with increasing concentrations of ABT263 for 72 h. Scale bar: 100 μm. (**D**) statistical analysis of living cells of three independent experiments is shown. (**E**) Analysis of cell apoptosis by flow cytometry with increasing concentrations of ABT263 after 48h treatment. (**F**) Statistical results of apoptotic cells of three replicates are also shown.

To confirm the successful induction of SnCs by IR, SA-β-gal staining and crystal violet staining were performed. Morphologically, IR-induced SnCs presented higher volume with an increased proportion of cytoplasm than that of normal chondrocytes under a light microscope. The portion of SA-β-gal positive cells was considerably higher in the IR SnC group than in the normal group. Crystal violet staining showed that there was a significant difference in cell proliferation between the two groups (Figure 2A). The gene expression of biomarkers of SnCs [25–27], CDKN1A (also known as p21) and CDKN2A, was analyzed by quantitative real-time polymerase chain reaction (RT-PCR). The results confirmed higher expression of CDKN1A and CDKN2A in IR-induced SnCs than in the control (Figure 2B). As shown in Figure 2C, IR-induced SnCs were efficiently eliminated by ABT263 and a few live cells were found in the group treated with 5.0 μM ABT263. Furthermore, a typical image of flow cytometry (FCM) analysis of Annexin V–propidium iodide (PI) staining showed that the percentage of apoptotic cells increased, which indicated that ABT263 eliminated SnCs by inducing cell apoptosis (Figure 2D).

### SA-β-gal-positive SnC accumulation in human OA articular cartilage and efficient elimination by ABT263 in micromass culture

**Figure 3:**
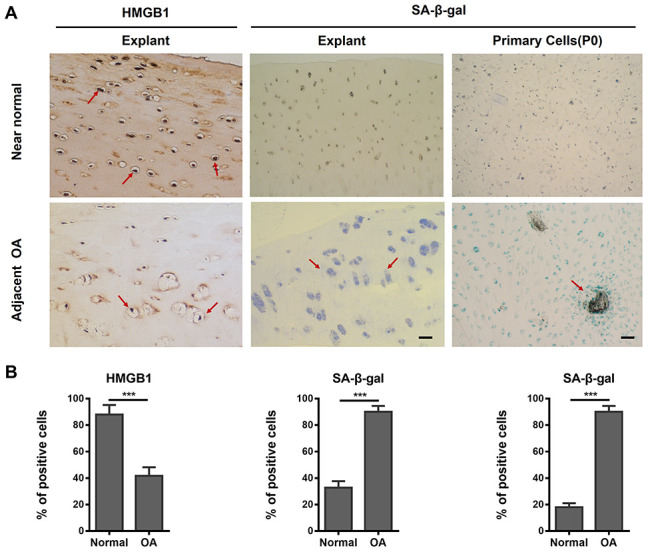
**The presence of senescent human chondrocytes (SnCs) in adjacent normal and advanced osteoarthritic articular cartilage.** (**A**) Representative images of HMGB1-positive non-SnCs (brown staining at red arrows, scale bars, 100 μm) by immunohistochemistry and SA-β-gal-positive SnCs expression on OA articular cartilage, OA chondrocytes (P0) and adjacent normal cartilage. (**B**) The statistical analysis of the percentage of SA-β-gal and HMGB1 positive cells from three independent experiments. Scale bar: 100 μm.

To further investigate the existence and accumulation of SnCs in OA cartilage tissue, we performed SA-β-gal staining and immunohistochemical staining for high-mobility-group box 1 (HMGB1)—an extracellular alarm signal, which is expressed in the nucleus prior to the secretion of SASP factors in cells undergoing senescence [28, 29]. For cartilage explants, stronger staining of HMGB1 was found in the adjacent normal group (OA cartilage with slight lesions in gross observation) than in the OA (severe damage in gross observation) group, yet a larger SA-β-gal positive region was observed in the OA group than in the adjacent normal group (Figure 3A). For chondrocytes, the staining results of SA-β-gal confirmed a considerably higher percentage of SnCs in primary OA chondrocytes than in the adjacent normal group (indicated in red arrow in Figure 3A). The results of quantitative analysis (an average of three different views) showed a significant difference between the two groups (Figure 3B).

**Figure 4:**
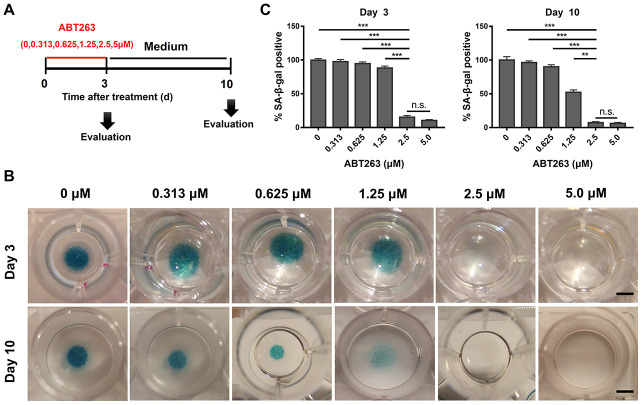
**Dose-dependent elimination of senescent cells in OA chondrocytes (P1) isolated from human OA tissue after ABT263 treatment for 72h.** (**A**) Schematic of the following experiment b–c. (**B**) SA-β-gal staining images shows a dose of 2.5 and 5.0μM effectively eliminated SA-β-gal positive senescent cells in OA chondrocytes (P1) by micromass culture in 48-well plates with increasing concentrations of ABT263 after different treatment time. Scale bar: 5 mm. (**C**) SA-β-gal positive cells were quantified 3 and 10 days following treatment with increasing concentrations of ABT263. Data are shown as mean ± standard deviation. N = 3 per group. **P < 0.01, ***P < 0.001 and n.s. (Not Significant); One-way ANOVA (Tukey’s multiple comparison test). N = 3 for 0, 0.313, 0.625, 1.25, 2.5, 5.0μM.

Micromass culture system was used to evaluate the concentration-dependent treatment effect of ABT263 on OA chondrocytes for 3 and 10 days. Figure 4A shows the schematic profile of screening experiments. The staining results revealed that SA-β-gal positive cells were potently cleared by ABT263 at 2.5 and 5.0 μM concentration compared with that at other concentrations (Figure 4B). Therefore, we suggest that 2.5 μM is the most appropriate concentration to efficiently eliminate SnCs, considering the cytotoxicity of ABT263 at 5.0 μM concentration. The quantitative data from three different samples, as shown in Figure 4C, also indicated that the percentage of SA-β-gal positive cells decreased in ABT263 concentration-dependent manner.

### Effect of ABT263 on OA chondrocytes in monolayer culture and clearance of SnCs via the apoptosis pathway

**Figure 5:**
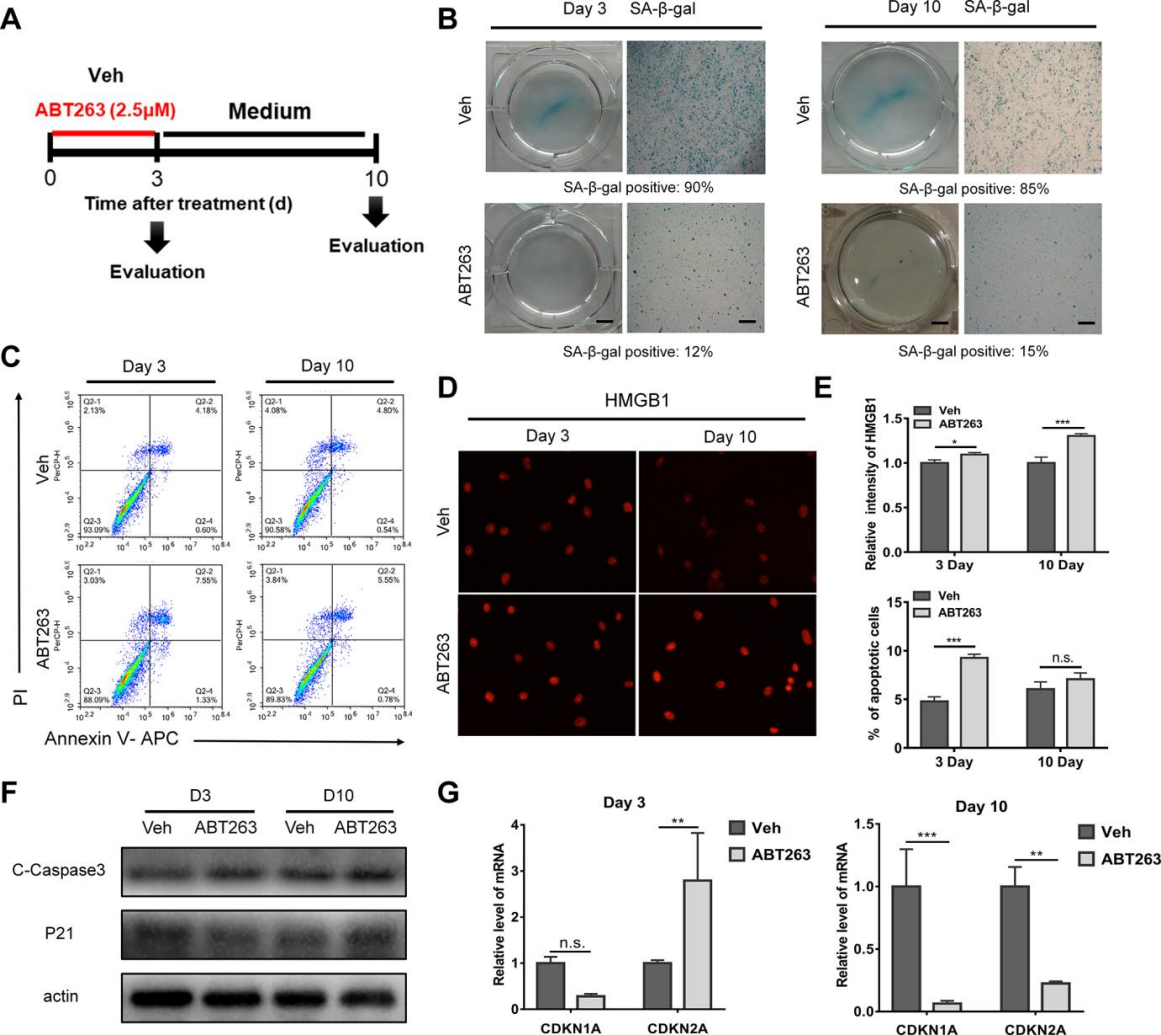
**A short - and long-term evaluation of the clearance effect of ABT263 on OA chondrocytes (P1) in monolayer culture.** (**A**) Schematic of the monolayer experiments in b–f. (**B**) Gross observation (left; scale bar: 5 mm) and microscope images (right; scale bar: 500 μm) of OA chondrocytes after treatment with 2.5μM ABT263 with SA-β-gal staining. (**C**) Representative flow cytometric plots measuring cell apoptosis from two independent experiments. (**D**) Immunofluorescence of HMGB1 in cultured OA chondrocytes at day 3 and day 10. Scale bar: 100 μm. (**E**) Statistical analysis of the percentage of apoptotic cells and fluorescence intensity of HMGB1 from three independent experiments are shown. (**F**) Western blot analysis of proteins level for c-caspase3 and p21 at Day 3 and Day 10. (**G**) qPCR analysis of mRNA levels for CDKN1A and CDKN2A (N = 3 for each group).

After preliminary screening, ABT263 at a concentration of 2.5 μM was chosen for the following experiments on OA chondrocytes isolated from human tissue in monolayer culture. The primary OA chondrocytes were seeded in 6-well plate at density of 2 × 10^5^ cells/ml. Treatment with ABT263 for 3 and 10 days, a relatively short-term and long-term assessment, was carried out separately as shown in Figure 5A. Firstly, SA-β-gal staining was performed to verify whether SnCs were selectively removed after a 3-day treatment period and the amount of SnCs was continuously decreased followed by another 7-day culture in vitro. Gross observation and typical images under a light microscope are shown in Figure 5B. The percentage of SnCs acquired from three different views declined from 90% to 12% within 3 days, but still remained approximately 15% after another 7 days of culture (Figure 5B). Annexin V-FITC/PI live–dead cell staining was then performed to measure the effect of ABT263 on apoptosis via flow cytometry. The results in Figure 5C showed that the percentage of total apoptosis on day 3 significantly increased in the ABT263-treated group compared with that in the vehicle group (from 4.78% to 8.88%), yet there was no statistical difference between the two groups on day 10 of evaluation from three independent experiments (Figure 5C, 5E). Furthermore, we determined the expression of HMGB1 by immunofluorescence at two-time points (3 and 10 days). As shown in Figure 5D, the fluorescent of HMGB1 underwent a relative enhancement after incubation with ABT263 for 72 h and the variation of fluorescent intensity of HMGB1 became more obvious between ABT263 group and vehicle group on day 10, which is consistent with the findings of previous study [22]. The statistical results from an average of three different views also demonstrated statistical distinction between the ABT263 and vehicle groups (Figure 5E). We then performed immunoblotting to analyze the protein expression of cleaved caspase-3 (cCasp3) and p21 in OA chondrocytes, which are involved in chondrocyte apoptosis during OA, on days 3 and 10 [30, 31]. The increased protein level of cCasp3 at the two time points and decreased expression of p21 on day 3 indicated that apoptosis was involved in the killing effect meditated by ABT263, although the p21 level displayed an increasing trend on day 10 (Figure 5F). The relative expression of *cdkn1a* and *cdkn2a* was determined by qPCR. As shown in Figure 5G, the decrease in *cdkn1a* expression showed no statistical significance and an unexpected increase in *cdkn2a* expression was found on day 3. Nevertheless, the expression of both genes considerably decreased on day 10 of treatment as expected. The incoherence between increased protein expression and decreased gene expression of p21 at day 10 suggested that the relation between mRNA and protein is not strictly linear, but has a more intrinsic and complex dependence. Complicated regulation mechanisms (involving translation and post-translational modification), acting on both mRNA and protein synthesis, affect the amount of expression on different level. Taken together, these results revealed that ABT263 could exert killing effect on SnCs in monolayer culture by inducing cell apoptosis and might have a sustaining influence on SnCs for a long period.

**Figure 6:**
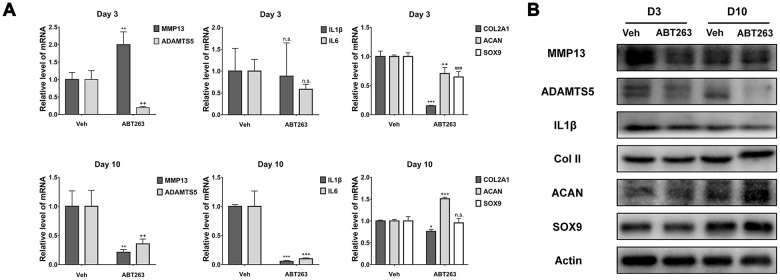
**Effect of ABT263 on OA chondrocytes in monolayer culture.** (**A**) qPCR analysis of mRNA expression for MMP13, ADAMTS5, IL1β, IL6, COLII, ACAN and SOX9 at Day 3 and Day 10 separately. (**B**) Immunoblotting analysis of protein level for MMP13, ADAMTS5, IL1β, COLII, ACAN and SOX9 at Day 3 and Day 10 separately. Data are shown as mean ± standard deviation. N = 3 per group.

To further estimate the role of ABT263 on the removal of SnCs in monolayer culture and the continuous effect it exerted, we investigated the mRNA and protein expression of SASP-related factors (MMP13, ADAMTS5, IL1β, and IL6) and chondrogenesis-related biomarkers (collagen II, ACAN, and SOX9), which are involved in the pathological processes of OA, using qPCR and western blotting [32]. As shown in Figure 6A, at the mRNA level, the expression of *ADAMTS5* was significantly decreased, whereas the expression of *MMP13* was increased, and no significant alteration was found in the IL1 and IL6 levels on day 3. However, the expression of all the above-mentioned genes was significantly reduced on day 10. Consistent with the mRNA expression change on day 10, ABT263 treatment downregulated the protein level of MMP13, ADAMTS5, and IL1β at the two time points, especially a significant inhibition of ADAMTS5 on day 10 (Figure 6B). Unexpectedly, the mRNA level of *COLII*, *ACAN*, and *SOX9* presented a decrease on day 3. The reason could be that due to the killing effect of ABT263, the remain cells underwent stress reaction at some extend, which was caused by the cellular debris or apoptotic products in the surrounding environment. Moreover, incubation with ABT263 enhanced the protein expression of ACAN on days 3 and 10, while little influence was observed on the protein expression of COLII and SOX9 (Figure 6B). Collectively, the above results revealed that ABT263 could reduce the protein expression of SASP-related cytokines and promote anabolism of aggrecan with long-term treatment.

### Evaluation for ABT263 treatment of OA chondrocytes in pellet culture: inflammation relief and chondrogenic phenotype promotion

**Figure 7:**
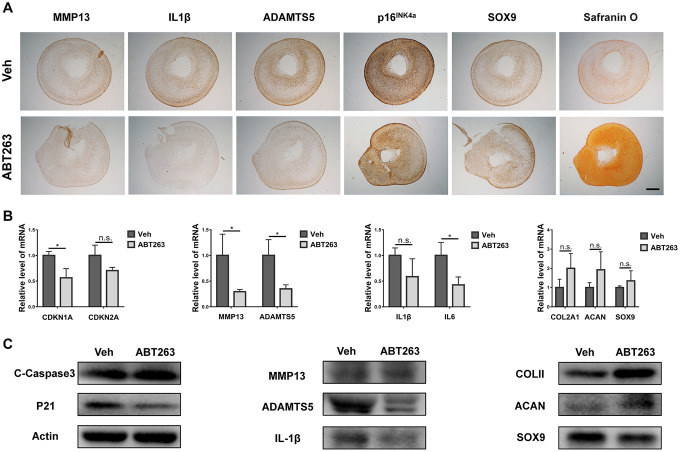
**Improvement of inflammation and promotion of proteoglycan anabolism.** (**A**) immunohistochemical analysis of senescence and inflammation-related markers and safranine O staining after 21-day pellet culture. Scale bar: 200 μm. (**B**) mRNA expression analysis by qPCR for CDKN1A, CDKN2A, MMP13, ADAMTS5, IL1β, IL6, COLII, ACAN and SOX9. (**C**) protein level analysis using Immunoblotting for C-Caspase3, p21, MMP13, ADAMTS5, IL1β, COLII, ACAN and SOX9. Data are shown as mean ± standard deviation. N = 3 per group.

Previous studies have reported that a similar senolytic agent (UBX0101) could support the maintenance of chondrocyte phenotype and formation of cartilage in pellet culture [22]. Therefore, we aimed to explore whether ABT263 could improve inflammatory microenvironment and chondrogenic differentiation. A 3D pellet culture system was used to evaluate the effect of ABT263 on chondrogenesis of human OA chondrocytes. After 21 days of growth in culture supplemented with chondrogenic medium, OA chondrocyte pellets were exposed to 2.5 μM ABT263 for 5 days. We then analyzed the mRNA and protein. expression of the senescence-related gene (*CDKN1A/p21* and *CDKN2A/p16*), inflammatory cytokines (IL1 and IL6), catabolism-related gene (*MMP13* and *ADAMTS5*), and chondrogenic markers (*COLII*, *ACAN*, and *SOX9*). The IHC staining results (Figure 7A) showed that the expression of MMP13, IL1β, and ADAMTS5 was considerably reduced after ABT263 treatment for 5 days. Meanwhile, ABT263 downregulated the expression of senescence-related gene *p16INK4a* and moderately upregulated the expression of *SOX9*. Notably, synthesis anabolism of glycosaminoglycan (GAGs) was greatly enhanced as observed by safranin O staining, representing an accelerated process of matrix synthesis in chondrogenesis (Figure 7A). At the mRNA level, accompanied with decreased expression of CDKN1A and CDKN2A, ABT263 reduced the *MMP13*, *ADAMTS5*, *IL1β*, and *IL6* mRNA levels and simultaneously increased the *COL2A1*, *ACAN*, and *SOX9* mRNA levels as shown in Figure 7B. Similarly, western blotting confirmed that the protein levels of IL1 and ADAMTS5 decreased considerably, and COLII and ACAN protein levels sharply increased, indicating enhanced extracellular cartilage matrix formation. Additionally, consistent with the immunoblotting results inmonolayer culture, increased cCasp3 protein level and reduced p21 expression verified that the apoptosis-related signal pathway might be involved in the SnC elimination process (Figure 7C). In brief, ABT263 could suppress the expression of inflammatory molecules and degradative enzymes and simultaneously promote cartilage matrix deposition in vitro.

### Post-traumatic OA was alleviated by intra-articular injection of ABT263 in the DMM rat model

**Figure 8:**
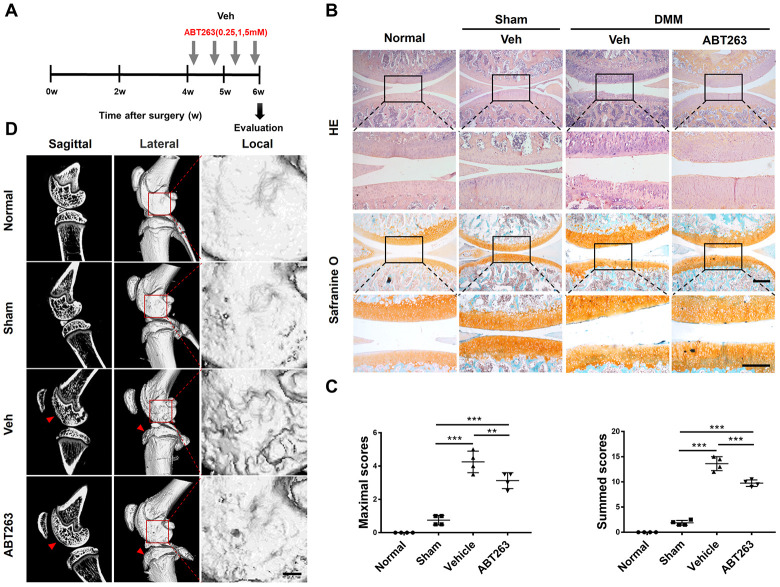
**Senescent cells clearance by treatment with ABT263 after rat DMM surgery attenuates the injury of post-traumatic OA.** (**A**) Schematic of the time course for the experiments in b–d. Male SD rat that underwent DMM surgery were injected intra-articularly every other day with vehicle (Veh) or ABT263 for two weeks and evaluated as indicated. (**B**) Representative images of Hematoxylin and eosin staining and safranin O and methyl green staining of the medial femoral condyle and medial tibial plateau in rat right knee. Scale bar: top: 500 μm; bottom: 100μm. (**C**) OARSI scores of the knee joints of rat with DMM or sham surgery. N = 4 per group. (**D**) Sagittal micro-CT and three-dimensional reconstruction images of the hind knee joint from different groups. ABT263 of 1.0 mM was corresponded to the results presented in B, C and D; Data are shown as mean ± standard deviation. Scale bar: 2 mm.

A previous study has demonstrated that SnCs developed and accumulated in p16-3MR transgenic mouse joints after injury induced by ACLT surgery by monitoring the expression of p16INK4a [22]. To further evaluate the effect of ABT263 on SnCs in vivo, DMM surgery was carried out in male SD rats to induce post-traumatic OA. To determine the most suitable concentration of ABT263 for in vivo experiment, ABT263 at different concentrations (0.25, 1, and 5 μM) was administered via intra-articular injection at four weeks after DMM surgery. Four intra-articular injections were administered within a period of two weeks, and then histological evaluation of cartilage structure and proteoglycan deposition in both experimental and contralateral joints was carried out by hematoxylin and eosin staining and safranin O/fast green staining at six weeks after surgery (Figure 8A). The representative images in Figure 8B demonstrated that the rats subjected to DMM surgery developed post-traumatic OA. Furthermore, more severe damage of cartilage integration on the tibia surface was observed in the vehicle group, while the ABT263 treatment group exhibited a relatively intact cartilage structure with a smoother tibia surface than that of the control group. Additionally, the OARSI scoring system including maximal and summed scores was used to quantitatively evaluate the degree of cartilage injury. Lower scores for the ABT263 group represented a milder cartilage lesion than that of the control group with higher scores, indicating the protective effect of ABT263 on the cartilage in vivo after injury (Figure 8C).

Furthermore, micro CT for bone scanning was carried out to investigate the subchondral bone damage after surgery. The representative images from sectional and 3D reconstructed views (Figure 8D) showed a collapsed

structure of subchondral bone in the anterior tibial plateau (red arrows). Additionally, locally amplified 3D reconstructed images of lateral femoral condyles displayed a relatively smooth surface in the ABT263-treated group, whereas osteophytes were observed in the same position in the vehicle group, which demonstrated that progressive damage of subchondral bone was prevented by ABT263 injection (Figure 8D).

**Figure 9:**
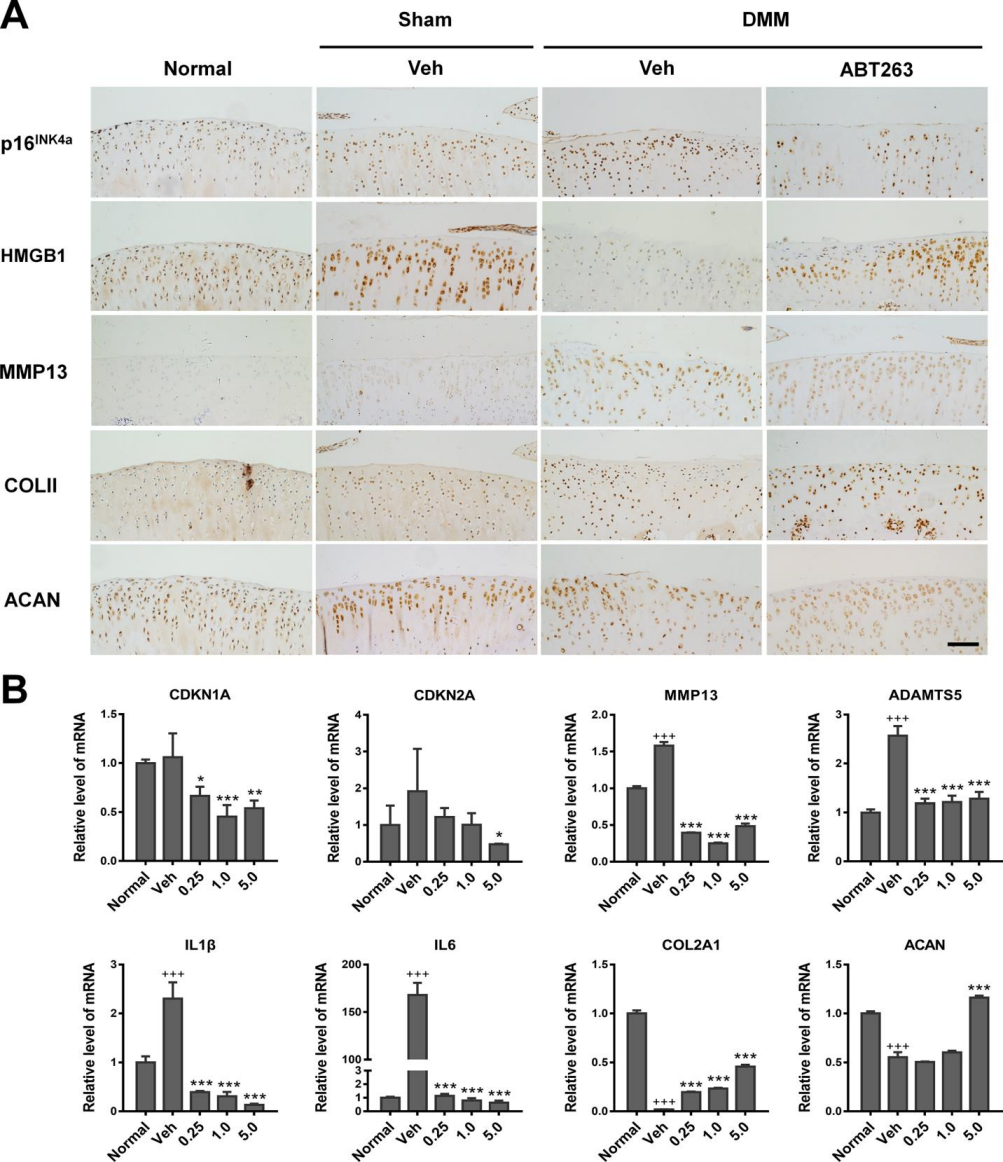
**Elimination of SnCs by ABT263 and alleviation inflammatory microenvironment in vivo.** (**A**) immunohistochemical analysis for expression of p16^INK4a^, HMGB1, COLII and ACAN of rat cartilage after 2 weeks intra-articular injection of ABT263. Result from ABT263 of 1.0 mM was presented. Scale bar: 100 μm. (**B**) mRNA level analysis using real-time qPCR for CDKN1A, CDKN2A, MMP13, ADAMTS5, IL1β, IL6, COLII and ACAN in the rat knee joint. Data are shown as mean ± standard deviation. N = 3 per group. +p<0.05 with respect to normal group; *p<0.05 with respect to vehicle group.

To acquire further insights into the effects of ABT263 on SnC clearance and cartilage matrix anabolism, we determined the expression of p16INK4a, HMGB1, MMP13, and COLII by IHC analysis. As shown in Figure 9, p16INK4a expression showed an apparent increase in the control group, which was partially inhibited by ABT263 injection. For HMGB1, the DMSO-treated surgery group displayed an evident decrease, which was rescued to a large extent by ABT263 injection. For MMP13, there was strong positive staining in the control group, yet a reduced expression was observed in the ABT263 treatment group. Moreover, COLII expression was weakened in superficial cartilage on the tibia side in the DMSO group. In the same position, ABT263 treatment resulted in an increase in COLII expression, followed by the control group. All quantitative results were obtained and analyzed from at least three different views of staining. In brief, the above results illustrated that the injection of ABT263 could prevent cartilage matrix degradation from post-traumatic OA and promote collagen deposition by eliminating SnCs in vivo.

Subsequently, we assayed the relative expression of genes highly involved in aging, SASP, and chondrogenesis processes, by RT-qPCR. As shown in Figure 9B, the mRNA levels of *CDKN1A*, *CDKN2A*, *MMP13*, *ADAMTS5*, *IL1β*, and *IL6* were upregulated in the vehicle group compared with those in the normal group, while an apparent reduction occurred in the expression of these genes after injection of ABT263 at all concentrations. This was consistent with the IHC results (Figure 9A). Meanwhile, the expression of anabolism markers (COLII and ACAN) decreased in the vehicle group, which was partially reversed by ABT263 injection. On the whole, ABT263 at 5.0 μM concentration demonstrated the most considerable effect on the removal of SnCs and anabolic synthesis of cartilage extracellular matrix among all the concentrations tested (Figure 9B). Taken together, ABT263 injection alleviated post-traumatic OA and might delay the progression of cartilage degeneration.

## DISCUSSION

With aging, cell senescence plays an important role in age-related cartilage degeneration and matrix degradation [33]. Targeting SnCs as an OA therapeutic strategy has been proposed recently, and eliminating SnCs by administering senolytic drugs has been demonstrated as an accessible approach [34]. The senolytic agent ABT-263, an inhibitor of Bcl-2, Bcl-xL, and Bcl-w, demonstrated senolytic activity on senescent human umbilical vein epithelial cells (HUVECs), IMR90 human lung fibroblasts, and murine embryonic fibroblasts (MEFs) [23]. In this study, we investigated the senolytic activity of ABT263 on senescent chondrocytes, and our results demonstrated that ABT263 reduced the production and secretion of SASP-related cytokines and alleviated the development of post-traumatic OA by selectively eliminating SnCs in the cartilage tissue. Moreover, we found that ABT263 could promote proteoglycan deposition in chondrocyte pellet culture, suggesting enhanced cartilage matrix anabolism after the removal of SnCs. It has been shown that the SA-β-gal activity correlates with OA severity [35], and a previous study reported that SA-β-gal positive staining was observed in only OA samples, but not in normal cartilage [17]. However, Martin and Buckwalter [36] showed the expression of SA-β-gal and decreased telomere length with age in chondrocytes from normal cartilage. In our study, SA-β-gal positive cells were found in explants and primary chondrocytes from both OA and the adjacent normal tissues, and the percentage of SA-β-gal positive cells in the OA group was considerably higher. In the CCK-8 assay, ABT263 showed a dose-dependent killing effect on IR-induced rat SnCs, while there was no obvious effect on the viability of OA chondrocytes. A possible explanation could be that with appropriate concentration, ABT263 only targets the senescent subpopulation without affecting the viability of non-SnCs.

In this study, the results of crystal violet staining showed a significant reduction in the proliferation of IR-induced SnCs. Cell senescence can, therefore, lead to tissue aging accompanied by aging-associated events, such as SASP [37]. It has been shown that SASP plays an important role in the development and progression of OA, associated with several upregulated cytokines (IL-1 and IL-6), growth factors (transforming growth factor β and epidermal growth factor), and matrix-degrading enzymes (MMP-3 and MMP-13) [12, 38, 39]. Interestingly, Farr et al. [40] and Jeon et al. [22] demonstrated that eliminating SnCs in the bone and cartilage can inhibit the expression of SASPs and proinflammatory secretome both in vitro and in vivo, resulting in increased cartilage formation and reduced bone loss [41]. Similarly, in our study, the expression of MMP13, ADAMTS5, IL1β, and IL6 was significantly inhibited in both monolayer culture and pellet culture. However, on day 3, in monolayer culture, there was an increase in the mRNA level of *p16* and *MMP13* and reduced gene expression of *COLII*, *ACAN*, and *SOX9*, possibly because large amounts of inflammatory factors and matrix-degrading enzymes were leaked into the extracellular microenvironment due to the cracking of the apoptotic cells within a short period [30, 42]. These data suggest that a transient ABT263 treatment might cause a reversible cell reaction of apoptotic products in surrounding normal cells, which resumes cell proliferation and anabolism after the removal of ABT263. It has been reported that there is a concomitant increase in the expression of the senescence-related gene including *p16*, *p21*, and *p53* in SnCs [12] and MSCs, in correlation with the presence of SA-β-gal [33]. In this study, we also found an upregulation of the mRNA expression of *p16* and *p21* in different batches of IR-induced SnCs. The expression of *p16* and *p21* was significantly decreased after ABT263 treatment, indicating a strong senolytic effect on SnCs.

Finally, we evaluated the outcome of intra-articular injection of ABT263 in a rat OA model. The histological staining results revealed that the vehicle group exhibited severe damage on superficial cartilage of the tibia side, while a relatively smooth cartilage surface was observed in the ABT263 treatment group, indicating that ABT263 injection might prevent superficial cartilage from matrix degeneration. Zhang et al. [43] reported that killing articular surface chondrocytes in mice by inducing autonomous expression of diphtheria toxin led to decreased cartilage damage, which suggested that superficial chondrocyte death does not directly affect cartilage damage in response to traumatic injury. Their findings imply that chondrocyte catabolism, rather than death, contribute to cartilage destruction following injury. In this study, SnCs in superficial cartilage were killed by ABT263 via apoptosis; consequently, chondrocyte catabolism was reduced near the cartilage surface, leading to decreased cartilage damage in the ABT263 group. Moreover, the local-amplified images from micro CT 3D reconstruction showed a smoother surface on the lateral view of the femur condyles in the ABT263 group that that in the control group, suggesting milder damage to the subchondral bone. These results imply that the severity of subchondral bone destruction has a correlation with cartilage damage degree in post-traumatic OA development and that ABT263 may play a protective role in both cartilage and subchondral bone.

There were some limitations to this study. First, we only evaluated the effect of ABT263 on cartilage and chondrocytes. The potential effects of ABT263 on other joint tissues, such as the synovial membrane and adipose tissues, also need to be addressed. Second, we performed intra-articular injections of ABT263 at four weeks after DMM surgery and harvested joint samples immediately after two weeks of injections; thus, only a relatively short-term efficacy on early phase of post-traumatic OA could be observed. However, the long-term effects of ABT263 need further investigation. Finally, the signaling pathways involved in ABT263-induced chondrocyte apoptosis remain to be clarified, and we intend to illustrate the detailed mechanism underlying the clearance effect of ABT263 on chondrocytes in future studies gradually.

The present study provides novel avenues associated with the clinical application of ABT263. Our results demonstrate that SnCs accumulated in human OA cartilage tissues and senescent chondrocytes in OA are selectively eliminated by ABT263 of suitable concentration through apoptosis. The reduced expression of SASP and promoted chondrogenic phenotype are observed in monolayer and pellet-cultured osteoarthritic chondrocytes. We further found that intra-articular injection of ABT263 attenuates the cartilage and subchondral bone damage in post-traumatic OA rat model. These findings provide evidence of the relevance of SnCs and inflammation in osteoarthritic cartilage, and also support the presence of SnCs and impact of senolytic treatment in OA. Direct targeting and killing of SnCs by senolytics provides a potential opportunity to mitigate OA from the source of the disease. Therefore, assessment of the effect of ABT263 on osteoarthritic chondrocytes provides a novel application in clinical therapies of OA by targeting SnCs.

## MATERIALS AND METHODS

### Cell isolation and culture

Human articular cartilage tissue was acquired from patients (average age 65 ± 5 years, n = 4) with advanced OA undergoing total knee arthroplasty from Center for Joint Surgery of Southwest Hospital. All human clinical samples were collected after informed consent and approval from the Ethics Committee of Southwest Hospital (Chongqing, China). The primary OA chondrocytes were harvested according to a previous study [44]. Cells were cultured in Dulbecco’s modified Eagle’s medium (DMEM; Gibco) containing 10% fetal bovine serum (Gibco) and 1% penicillin/streptomycin (Gibco) in an atmosphere of 5% CO_2_. Culture medium was changed every 2 or 3 days. The isolation and culture of rat primary chondrocytes were conducted using the same protocol above. Both types of chondrocytes were expanded in vitro and the primary or passage 1 were used in the following experiments.

### Pellets and micromass culture

The preparation of the pellets was according to a previous study by Johnstone et al. (1998) [45]. Cells were harvested at density of 5 × 10^5^ cells/ml, suspended in 500 μl chondrogenic medium, centrifuged at 500 × g for 5 min in 15-ml polypropylene conical tubes. Pelleted cells were incubated at 37 °C under 5 % CO2 and the caps were loosened to permit gas exchange. Within 48 h of incubation, the sedimented cells formed a spherical aggregate at the bottom of each tube. The chondrogenic medium consists of high-glucose DMEM supplemented with 2% fetal bovine serum (Gibco), 10 ng/ml recombinant human transforming growth factor-b3 (TGF-b3; Peprotech), 100 nM dexamethasone (Sigma), 50 μg/ml ascorbic acid 2-phosphate (Sigma), 1 mM sodium pyruvate, 40 μg/ml proline and 1% ITS-A supplement (Gibco). The medium was changed every 3 days a week and pellets were harvested on days 21. For micromass culture, in brief, cells were harvested and resuspended in chondrogenic medium at 1 × 10^7^ cells/ml. Droplets (20 μl) were carefully placed in a 24-well plate. Cells were allowed to adhere at 37 °C for 2 h, followed by the addition of 500 μl chondrogenic medium and the medium was changed every 3 days a week.

### Ionization irradiation on chondrocytes

To induce senescent cells in vitro, primary rat chondrocytes at 80% confluence were exposed to X-ray ionization irradiation under the circumstance of 300KV, 4 mA for 20 min (total irradiation dose 10 Gy). After irradiation, the cells were cultured for 3 days and then passaged. Then, these cells were cultured for another 7 days and used for SA-β-gal staining and qPCR analysis to verify senescence.

### Cell viability assays

Cell Counting Kit–8 (CCK-8) analysis was used to assess cell viability after ABT263 treatment. In short, cells at passage 1 were seeded into 96-well plate and cultured for 24 h. Then cells were treated with vehicle (0.1% DMSO or PBS) or ABT263 at increasing concentrations. After incubation for 24h, 48h, and 72h, absorbance at 450 nm wavelength was measured using the Cell Counting Kit–8 (Dojindo).

### Crystal violet staining

Cell proliferation was assessed using the crystal violet assay. IR-induced rat senescent chondrocytes and rat normal chondrocytes were seeded in a 6-well plate at 1.0×10^4^ cells/well and then cultured in DMEM medium containing 10% FBS for 10 days. After that, the cells were stained with a staining solution (0.5% crystal violet (Sigma-Aldrich, 61135) in 30% ethanol and 3% formaldehyde) for 15 min at room temperature, washed three times with PBS, and dried. Cell morphology was observed under an inverted microscope (magnification, ×40, ×100).

### SA-β-galactosidase staining

SA-β-gal staining was done using a Senescence β-Galactosidase Staining Kit (Cell Signaling Technology #9860) according to the manufacturer’s instructions. Briefly, cells in 6-well plate were fixed with 4% paraformaldehyde and then incubated with staining solution overnight. SA-β-gal-positive senescent cells were stained blue. Staining for micromass culture in 24-well plate was performed using the same staining kit and procedure as mentioned above. SnCs were recognized as blue-stained cells under light microscopy. Percentage of SA-ß-gal-positive cells were counted in three random fields per culture dish using software ImageJ according to manufacturer’s instruction. For quantitative statistics in micromass, we used Image Pro Plus 6.0 to measure the percentage of the preselected stained area referring to the whole area in the three random fields for each group and calculated relative to the vehicle group.

### Histology

Pellets and rat joints were fixed in 4% paraformaldehyde overnight, dehydrated in increasing concentrations of ethanol and embedded in paraffin. Sections (5 μm) were cut from the paraffin blocks and applied to glass slides. The sections were stained for hematoxylin-eosin (H&E) and safranin O (0.1%) (Sigma-Aldrich) according to a previous study [46], and the specimens were then mounted and observed under light microscope.

### Immunohistochemistry

For immunohistochemical staining, the experiment was performed using SP-9000 Histostain-Plus kits (Zsgb Bio, China) according to the manufacturer’s protocol. Briefly, endogenous peroxidase activity was quenched with 3% (v/v) hydrogen peroxide in methanol, and then antigen retrieval was done via 0.1% trypsin, followed by blocking process with goat serum for 60 min. Sections were incubated overnight at 4 °C with the following primary antibodies: p16^INK4a^ (1:500; Abcam, ab54210), MMP-13 (1:200; Abcam, ab39012), type II collagen (1:300; Abcam, ab34712) and HMGB1 (1:500; Abcam, ab18256). Antibodies were diluted in 3% BSA dissolved in PBST with 2.5% Triton X-100. After rinsing with PBS for three times, sections were incubated with homologous biotinylated secondary antibody and horseradish peroxidase-conjugated streptavidin-biotin. The total number of positively stained cells on average 3 views of each section were counted using Image-Pro Plus version 5.1 software (Media Cybernetics) for histomorphometric measurements. The percentage of positive cells was calculated and the relative fold change was determined.

### Immunofluorescence

For immunofluorescence assay, human OA chondrocytes were firstly fixed in 4% paraformaldehyde, followed by permeabilization with PBS containing 0.25% Triton X-100 for 15 min and then blocked with 5% BSA containing 0.25% Triton X-100 for 60 min at room temperature. Then the cells were incubated overnight at 4 °C with primary antibodies against protein HMGB1 (1:100; Abcam). After rinse for three times with PBS, the cells were then incubated with goat anti-rabbit IgG (H&L) conjugated with Alexa Fluor 594 (1:300; Zsgb Bio, China). After rinse for three times, the nuclei were counterstained with DAPI for 3 min. Images were taken by fluorescence microscopy (Carl Zeiss, USA). Total cells were counted in five random fields per culture dish and percentage of HMGB1 positive cells were determined.

### Flow cytometry analysis

For Annexin V/PI analysis, cultured chondrocytes were trypsinized and floating cells in medium supernatant were collected, followed by centrifugation and rinse with PBS. Then cells were stained with the Annexin V/PI apoptosis detection kit APC according to the manufacturer’s instructions (BD Biosciences). Finally, samples were detected using BD Accuri C6 flow cytometer and data were analyzed by FlowJo X 10.0 software (Becton Dickinson).

### Real-time polymerase chain reaction (RT-PCR)

To extract tissue RNA of rat knee cartilage, the hindlimbs that underwent surgery and contralateral controls were dissected and collected in RNAlater™ Stabilization Solution (Thermo Scientific) for total RNA extraction. Rat knee cartilage was shattered using large-scale tissue pulverizer. As for mRNA detection, the cells were lysed and total RNA was extracted from chondrocytes using TRIzol Reagent (Invitrogen, Thermo Scientific). For chondrocyte pellets, Universal RNA Extraction Kit (Takara, Japan) was used for RNA extraction. Then total RNA was reversely transcribed to Complementary DNA (cDNA) using PrimeScript™ RT reagent Kit with gDNA Eraser (Takara, Japan) following the manufacturer’s protocol. The real-time PCR procedure was performed on CFX96 Touch™ Real-Time PCR Detection System (Bio-Rad, America) using TB Green® Fast qPCR Mix (Takara, Japan) according to the manufacturer’s recommendation. Each sample was prepared in triplicate for this experiment. The involved primer sequences are enlisted in Supplementary Table 1. All results were normalized to that for ß-actin. Relative expression level of all genes was calculated using the ΔΔCT method, in which ΔCT was calculated using the ß-actin reference gene. ΔΔCT was calculated relative to the normal group in the in vivo studies and relative to vehicle samples in the in vitro studies.

### Immunoblotting

Chondrocytes in monolayer culture and pellet culture were harvested, washed twice with pre-cooling PBS and lysed with RIPA buffer (Beyotime Biotechnology, China) containing PMSF (Beyotime, China) and phosphatase (Sigma, P5726) inhibitor mixture according to the manufacturer’s instructions. The total protein concentration was determined using a BCA protein assay kit (Beyotime, China). Proteins were separated by 10% SDS–PAGE and then transferred onto PVDF membranes. The membranes were blocked with 5% BSA in TBST for 1 h and incubated overnight at 4 °C with primary antibodies specific for cleaved caspase-3 (1:1,000; Affinity Biosciences, AF7022), p21 (1:1,000; Affinity Biosciences, AF6290), ß-actin (1:200; Santa Cruz Biotechnology, sc-47778), Collagen II (1:1,000; Abcam, ab34712), Aggrecan (1:100; Abcam, ab3778) and SOX9 (1:1,000; Abcam, ab185230). After incubation with primary antibodies and rinse with TBST, the membranes were incubated with horseradish peroxidase-conjugated goat anti-rabbit secondary antibodies (1:10,000, Zsgb Bio, ZB-2301) and goat anti-mouse secondary antibodies (1:10,000; Zsgb Bio, ZB-2305) and protein bands were detected using a Western ECL Substrate (Invitrogen, Thermo Scientific) for chemiluminescence with X-ray film.

### Surgically induced OA rat model

All animal experiments were in accordance with the Guide for the Care and Use of Laboratory Animals and were approved by Animal Research Committee of Third Military Medical University (Army Medical University). Destabilization of the medial meniscus (DMM) and sham surgery were performed on male SD rat aged 4-6 weeks. Animals were undertaken general anesthesia with 3% isoflurane, and hindlimbs were shaved and prepared for aseptic surgery. For the sham surgery, the knee articular cavity was exposed following a medial incision, and then the surgical incision was closed with sutures. At week 6, the rats were euthanized, and the knee joints were then harvested for histologic staining, μCT scanning, and mRNA analysis.

### Histologic analysis

Histological assessment of the joint was performed using the Osteoarthritis Research Society International (OARSI) scoring system [47, 48] through blinded graded observations by two observers. Histologic alterations in the medial tibial plateau and medial femoral condyle of knee joints were scored on a scale of 0–6 according to the recommendations of OARSI. The summed score and maximal score were taken to describe and estimate the severity of cartilage degeneration (sum of the 4 highest scores in all sections).

### In vivo micro-computed tomography (micro-CT) scanning

A type of bench-top cone-beam animal scanner (Quantum FX μCT, Perkin Elmer, USA) was used to analyze the hind knee joints of each rat. All specimens were scanned with the following parameters: 90 kV/160 μA, pixel size of 148 μm, and a 0.5 mm aluminum filter. The ABT263-treated knees and the contralateral controls were scanned separately. After scanning, a total of 512 cross-sectional slices were generated and collected, and then three-dimensional (3D) reconstruction of each sample was accomplished.

### Statistics

The data in this study displayed a normal variance. The investigators were not blinded to allocation during experiments and outcome assessment. The data were analyzed by analysis of variance (ANOVA) using GraphPad Prism 7 from GraphPad Software (San Diego, CA). In case that ANOVA justified post hoc comparisons between group means, these were conducted using Neuman-Keuls or Tukey’s multiple-comparisons test. Statistical significance was determined with P < 0.05.

## Supplementary Material

Supplementary Figure 1

Supplementary Table 1
