# Supplementary material for: Navitoclax (ABT263) reduces inflammation and promotes chondrogenic phenotype by clearing senescent osteoarthritic chondrocytes in osteoarthritis
Source: Aging (Albany NY). 2020 Jul 1;12(13):12750–70. doi: 10.18632/aging.103177 (PMC7377880; doi:10.18632/aging.103177)
Supplement: Supplementary Figure 1 [file aging-12-103177-s002..pdf]

SUPPLEMENTARY FIGURE

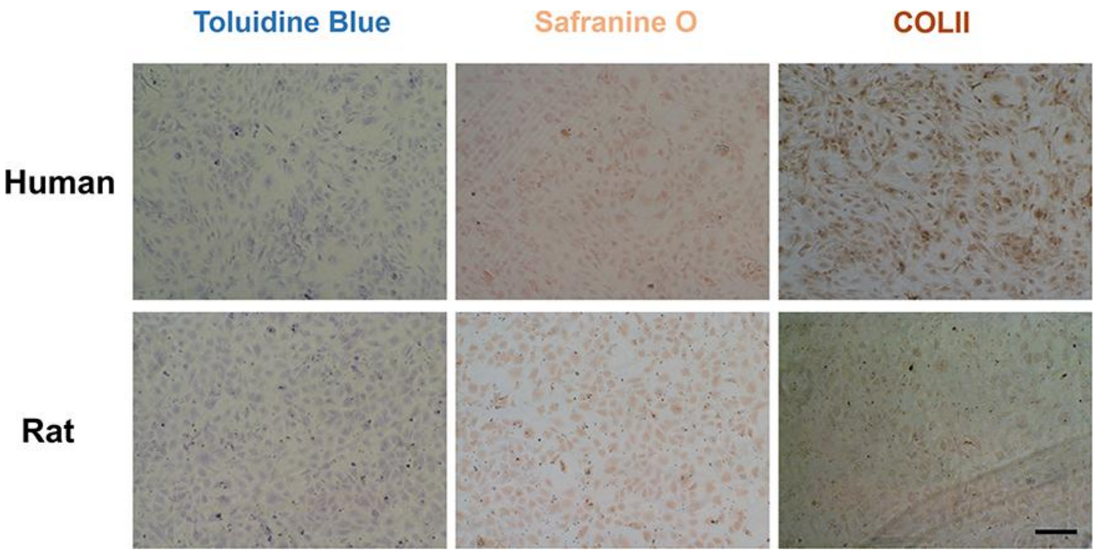

Supplementary Figure 1. Histological and immunohistochemical staining analysis for COLII and proteoglycan deposition in P1 chondrocytes derived from the TKA surgery patients and SD rat knees. Scale bars: 200 µm**.**
