# Supplementary material for: Navitoclax (ABT263) reduces inflammation and promotes chondrogenic phenotype by clearing senescent osteoarthritic chondrocytes in osteoarthritis
Source: Aging (Albany NY). 2020 Jul 1;12(13):12750–70. doi: 10.18632/aging.103177 (PMC7377880; doi:10.18632/aging.103177)
Supplement: Supplementary Table 1 [file aging-12-103177-s001..pdf]

SUPPLEMENTARY TABLE

Supplementary Table 1. Primers sequences used for real-time polymerase chain reaction (RT-PCR)

Gene (human)	Forward primer (5'-3')	Reverse primer (5'-3')	Product size (bp)
CDKN1A	TCCAGCGACCTTCCTCATCCAC	TCCATAGCCTCTACTGCCACCATC	108
CDKN2A	ACACCGCTTCTGCCTTTTCAC	TGAAGTCGACAGCTTCCGGA	119
MMP13	AATTAAGGAGCATGGCGACTTCTACC	TGGAGTGGTCAAGACCTAAGGAGTG	197
ADAMTS5	TGGCCTCTCCCATGACGATT	GCTTTCGTGGTAGGTCCAGC	188
IL1B	TGGCTTATTACAGTGGCAATGAGGATG	TGTAGTGGTGGTCGGAGATTCGTAG	138
IL6	ATTCAATGAGGAGACTTGCCTGGTG	ATCTGCACAGCTCTGGCTTGTTC	120
COL2A1	GGAGCAGCAAGAGCAAGGAGAAG	TGGACAGCAGGCGTAGGAAGG	138
ACAN	TCCTGGTGTGGCTGCTGTCC	TCTGGCTCGGTGGTGAACTCTAG	93
SOX9	CACACGCTGACCACGCTGAG	GCTGCTGCTGCTCGCTGTAG	100
β-ACTIN	AAGGTGACAGCAGTCGGTT	TGTGTGGACTTGGGAGAGG	195

Gene (rat)	Forward primer (5'-3')	Reverse primer (5'-3')	Product size (bp)
Cdkn1a	TCCTGGTGATGTCCGACCTGTTC	GCGGCTCAACTGCTCACTGTC	85
Cdkn2a	GCTGGATGTGCGCGATGCC	CAGAAGTTATGCCTGTCGGTGACC	141
Mmp13	CATACTACCATCCTGTGACTCTTGCG	CCACATCAGGCACTCCACATCTTG	171
Adamts5	TCTGCCTGCAAGGGAAATGT	CAATGGCGGTAGGCAAACTG	142
Il1b	GACAAGAGCTTCAGGAAGGCAGTG	CACACTAGCAGGTCGTCATCATCC	176
IL6	ATTCAATGAGGAGACTTGCCTGGTG	ATCTGCACAGCTCTGGCTTGTTC	120
Col2a1	ACGCTCAAGTCGCTGAACAACC	TCAATCCAGTAGTCTCCGCTCTTCC	131
Acan	AGTGAACAGCATCTACCAAGACAAGG	GAGTCATTGGAGCGAAGGTTCTGG	99
Sox9	GCGAGCAGCAGCAGCACTC	TCTGGTGGTCGGTGTAGTCATACTG	117
β-Actin	GCAGGAGTACGATGAGTCCG	ACGCAGCTCAGTAACAGTCC	74
